# Editorial: Computers and Games for Mental Health and Well-Being

**DOI:** 10.3389/fpsyt.2018.00141

**Published:** 2018-04-13

**Authors:** Yasser Khazaal, Jérôme Favrod, Anna Sort, François Borgeat, Stéphane Bouchard

**Affiliations:** ^1^Department of Psychiatry, University of Geneva, Geneva, Switzerland; ^2^Geneva University Hospitals, Geneva, Switzerland; ^3^Research Centre of the Montreal University Institute of Mental Health, Montreal, QC, Canada; ^4^La Source, School of Nursing Sciences, HES-SO University of Applied Sciences and Arts of Western Switzerland, Lausanne, Switzerland; ^5^University of Barcelona, Barcelona, Spain; ^6^PlayBenefit, Barcelona, Spain; ^7^Department of Psychiatry, University of Montréal, Montreal, QC, Canada; ^8^Department of Psychoeducation and Psychology, Université du Québec en Outaouais, Gatineau, QC, Canada; ^9^Centre Intégré de Santé et de Services Sociaux de l'Outaouais, Gatineau, QC, Canada

**Keywords:** internet treatment, virtual reality, serious games, augmented reality, smartphone app, cognitive remediation, cognitive behavior therapy

## Introduction

Recent years have seen major developments in the computer and game industry. Tools such as games, virtual reality, and applications for smartphones may foster learning, enhance motivation, promote cognitive and behavioral change, support psychotherapy, favor empowerment, and improve cognitive functions. Games and computer design share an opportunity for creativity and innovation in helping to specifically build and assess preventive or therapeutic tools.

The research topic computers and games for mental health and well-being illustrates some of the developments in the field across 34 papers written by 188 authors around the world. Overall, close to 30% of the papers consist of clinical studies, including two controlled trials; 20% are reviews that describe the potential of various technologies and applications; and about 50% express scientists' opinions, innovative perspectives, and thoughts about the use of computers and games. A scan of the research interests covered by this Research Topic reveals an important focus on clinical populations (at least 24 papers), especially schizophrenia and related psychotic disorders (nine papers), as well as significant coverage of serious gaming (seven papers), augmented or virtual reality (six papers), apps and smartphones (five papers), web-based applications (three papers), and much more. The present editorial aims to briefly summarize the contribution of the published papers.

## Prevention of mental health disorders

Prevention of mental health disorders with e-health- and m-health-related tools was explored by some of the topic authors (Ebert et al.; Baños et al.). Ebert et al. reviewed the evidence for the effectiveness of such Internet and mobile interventions in preventing the onset of several mental health disorders. In consideration of the limited number of randomized controlled trials related to such a question, it was not possible to draw any definite conclusion from the review. However, reports related to the prevention of depression showed promising findings.

Baños et al. reviewed the evidence specifically related to the impact of positive online interventions that aim to promote well-being and resilience in the adolescent population. The paper discusses the potential of such interventions for mental health promotion. Despite the promising rationale of these interventions, the authors concluded that more controlled studies are needed with long-term follow-up (Ebert et al.; Baños et al.).

## Serious games

In a perspective article, Desseilles discussed some of the paradoxes (i.e., using a game for a serious issue), opportunities (i.e., for learning and communication), and challenges (i.e., misuse and abuse-related risks) offered by games in relation to mental health. Fleming et al. explored the current status and promising directions for the development of games and gamification for mental health.

Some authors argued that games have the potential to increase the impact of Internet-based interventions by improving their reach and engagement potential. In their reviews, they detailed some of the main engagement mechanisms from games reported in the health literature, as well as from studies related to Internet gaming and Internet gaming disorders [1, 2]. They make the point that the ubiquity and variability of the possible game designs (exergame biofeedback, cognitive training, etc.) would help to train or foster specific change mechanisms adapted from traditional evidence-based interventions. According to these authors, the number of trials in the field is still limited. In particular, only a few trials included comparisons between game-based and non-game-based interventions [3].

Lau et al. however, conducted a systematic review and meta-analysis of randomized controlled trials related to the effectiveness of serious games for the treatment of mental disorders. The review included studies that involved a total of 674 participants treated for different mental conditions (depression, alcohol use disorder, etc.). A meta-analysis of nine studies revealed a positive moderate effect on symptoms favoring games over no-intervention control groups.

## The naturalistic uptake challenge

In a paper issued by an international Collaboration On Maximizing the impact of E-Therapy and Serious Gaming (COMETS), Fleming et al. highlighted a gap between the positive results of controlled studies and difficulties in uptake and adherence outside of trials. In response to the reach and engagement challenge, the COMETS group proposed a paradigm change in e-health that focuses on four pillars: (1) increasing user involvement and user-centered approaches in the field, (2) increasing emphasis on engagement (including via gaming), (3) increasing collaboration and data sharing, and (4) rapid testing and implementation.

De Beurs et al. described and discussed different methods that, consistent with the proposed COMETS paradigm, aim to increase end-user uptake in the development of web-based mental health interventions. Regarding improved uptake, Thorens et al. discussed the possible lessons learned from the success of massively multiplayer online role-playing games (MMORPGs) and some of the MMORPG mechanisms that could be extrapolated in designing games for health interventions. Finally, Sort and Khazaal proposed six tips that aim to improve game design processes and possibly increase the success of games for health in terms of reach, engagement, and clinical outcomes.

## Sensor-related technologies

Several authors reported on the integration of sensor-related technologies as possible tools to predict further emotional or behavioral modifications. One may assume that such tools would be helpful in increasing the rate and efficacy of early-delivered interventions.

Sun et al. compared heart rate variability (HRV) during a mental task in drug-naïve patients who had a major depressive disorder (MDD) with HRV in healthy controls. They concluded that HRV indices measured during a mental task are a promising tool for screening patients with MDD. In another pilot study, this time with patients who have intellectual disabilities and psychiatric disorders, Palix et al. showed promising results on the possibility of predicting clinical agitation from a change in HRV. Winslow et al. described the development of a classifier of real-time physiological stress (based on electrodermal and cardiovascular inputs) in a healthy population. In a pilot trial, they compared the addition of the classifier associated with an mHealth app intervention for stress management to the use of cognitive behavioral therapy (CBT) alone. The addition of the classifier to the app intervention was associated with greater improvement in measures of stress and anxiety, showing promising results that are to be confirmed in further studies.

## Smartphone apps

Benarous et al. reported a study protocol that aims to offer ecological momentary assessment and interventions through a smartphone app to adolescents with substance use and comorbid severe psychiatric disorders. The patients will also be assessed through clinical interviews at a 1-year follow-up. On the one hand, the study will increase our knowledge about the acceptability of such apps, and on the other, it may provide important inputs after 1 year of evolution in psychiatric and substance use-related symptoms among adolescents.

Monney et al. described the theoretical background of a smartphone app for cannabis users (“stop-cannabis”). They further reported the results of a satisfaction survey carried out among ~500 app users, showing high involvement, a good level of satisfaction, and a high level of perceived usefulness. The results are promising, considering the moderate-to-high level of cannabis dependence reported by the participants. Previous studies showed the ability of e-health interventions to appeal to people with moderate or severe dependence [4]. The self-selected [5] and non-randomized nature of the sample in the survey cannot, however, allow more conclusive statements about the app's efficacy. Benarous et al. planned to use the “stop-cannabis app” in their study, which may provide more evidence about such interventions. Similarly, Zhang and Ho illustrated in their perspective paper that recent developments in smartphone app-related technologies will open new prevention and treatment avenues for addictive disorders.

Van Singer et al. assessed the content quality, interactivity, and self-help features of the panic disorder smartphone apps available at Google Play Store. The authors adapted the tools used for the assessment of health-related websites to the smartphone apps [6–8]. They further developed a specific self-help assessment tool that was based on the adaptation of a panic disorder CBT into app format. As shown in other studies on the assessment of apps for different mental health disorders using the same [9] or different methodologies [10], the results led the authors to conclude that most of the assessed apps were of poor quality.

The findings on smartphone apps, as shown by other authors [9–12], highlight a gap between the wide availability of health-related smartphone apps in the market and lower levels of evidence-based conceptions of such tools.

## Computer-supported treatments

Zidani et al. reported on the effects of a computerized, masked, priming-based intervention called Augmentation of Psychotherapy through Alternative Preconscious Priming (APAP) [13]. The intervention was offered to eight treatment-resistant patients with social anxiety disorder or generalized anxiety disorder. In this case series, the authors found promising improvements in quality of life and anxiety symptoms and related beliefs.

Demily et al. described “Cognitus & Moi,” a computer-based cognitive remediation program for children with intellectual disability. Cognitus & Moi is based on a metacognitive strategy specifically dedicated to the training of attentional and visuospatial areas. The tool includes a variety of exercises and may offer individualized training according to the specific needs of the patient.

## Virtual reality and augmented reality

Six of the papers included in the research topic focused on virtual therapy. In their conceptual design paper, Ben-Moussa et al. proposed the DJINNI solution. This technology- and gamification-supported exposure therapy paradigm combines virtual reality and wearable augmented reality to complement traditional *in vivo* exposure in the treatment of social anxiety disorder. One aspect of significant interest in the proposed system is the possibility of an augmented reality system that interprets and guides the user during *in vivo* exposure exercises in the absence of a therapist. The data collected with this system could be used to improve and adapt virtual reality scenarios for use in exposure exercises.

The work of Urech et al. was also about the use of virtual reality for patients with social anxiety disorder. The paper described a proof-of-concept and pilot study on a new use of virtual reality. The originality of the tool lies in its ability to modify attentional biases, instead of using virtual reality, to expose users until they reach extinction of the feared response. The preliminary results suggest sufficient improvements in attention bias and social anxiety to argue for further assessments of such a promising technique with more robust clinical trials.

Addressing another severe mental disorder, compulsive hoarding, St-Pierre-Delorme et al. showed in a pilot control trial the potential benefits of adding a virtual reality component in which objects belonging to the patients can be integrated in the virtual environment. They recruited 14 adults with severe problems in accumulating objects without being able to throw them away and randomly assigned them to a virtual reality-based treatment in which they would discard common household objects that belonged to them (experimental treatment) or did not belong to them (control condition). The results were promising, encouraging the authors to pursue this line of research with a larger sample.

Working with a patient with obsessive-compulsive disorder, Laforest et al. also provided preliminary support for the use of a modified CBT treatment, focusing on the use of virtual reality to conduct exposure exercises related to fear of contamination. They used a single-case design with multiple baselines across subjects, combined with time-series analyses, a classic approach for demonstrating the impact of a new treatment before conducting randomized control trials.

Bouchard et al. presented another innovation related to the use of virtual reality, this time for the treatment of gambling disorder. The paper reported three successive studies about the development, usefulness, and safety of virtual reality environments designed to induce the desire to gamble. The authors considered these preliminary steps requirements before dedicating several therapy sessions to immersion in virtual reality in which psychotherapists would help pathological gamblers by applying therapy techniques while the gamblers were emotional and in a state of craving.

Contrasting with other virtual reality studies focusing on the treatment of a specific disorder, Riva et al. explored a transforming experience paradigm supported by augmented and virtual reality. From a solid understanding of the science behind their ideas and the observation that people are not always able to implement desired behavior change, the authors argue in favor of the use of augmented and virtual reality technologies, together with the feeling of presence and emotional engagement. They propose fostering change and transformative experiences by increasing perceived self-efficacy and self-reflectiveness and by structuring or altering bodily self-consciousness.

## Schizophrenia and related disorders

The large number of papers (9) on schizophrenia and related disorders is a good illustration of how clinicians and researchers in the field can improve the use of computers and games in the treatments being offered to patients.

Four papers concerned the development and evaluation of computerized programs for the remediation of cognitive deficits in schizophrenia. Amado et al. presented a pre-post pilot study of a virtual reality game to train attention, memory, and planning. The game simulates a town in which the participants navigate and plan actions that they have difficulty carrying out in real life. The pilot study of 12 interactive 90-min weekly sessions with this virtual reality game showed improvement in participants' attention and memory. Demily et al. presented a partially computerized program to improve impairments in social cognitive processes. The computerized program allows participants to develop social cognitive abilities in a simulated environment. The authors tested their program through single-case studies. Their results showed an improvement in the targeted processes in theory of mind and attributional styles. Gaudelus et al. measured the efficacy of a computerized program called GAÏA, which focuses on facial emotion recognition processes. Their randomized controlled trial compared GAÏA to another cognitive remediation program. The study showed a significant improvement in facial emotion recognition performance in both groups, with a significantly larger effect in the GAÏA arm. Vianin underlined the importance of not limiting cognitive remediation to the single use of computerized exercises. According to this author, cognitive remediation should involve the training of metacognitive skills throughout the intervention, as well as going back and forth between the computerized remediation training of cognitive deficits to real-life exercises to promote the generalization of learning.

Two papers examined online interventions. Thomas et al. developed a website that can be used on a tablet computer by mental health workers to structure therapeutic discussions about personal recovery. They tested the feasibility and acceptability of a low-intensity intervention with 10 participants. All participants improved their personal recovery, as measured by the Questionnaire for the Process of Recovery, and stated that they would recommend the interventions to others. Rehm et al. reviewed the literature on the use of avatars to facilitate online communication between clients and therapists. Their narrative review suggests that avatars serve several functions in facilitating treatment engagement through a virtual therapeutic alliance, which reduces communication barriers, promotes treatment seeking, promotes expression and exploration of self, and enables therapists to control treatment stimuli.

Three papers focused on the use of games or playful interventions to reduce symptoms by improving physical activity. Leutwyler et al. used a video game to improve physical activity in older adults with schizophrenia. Twenty participants played various fitness video games for 30 min once a week for 6 weeks. The results indicate a significant increase in frequency of self-reported vigorous physical activity, as well as a non-significant increase in number of steps taken and a reduction in sedentary hours. Khazaal et al. compared Michael's game with treatment as usual in a randomized controlled trial. The study included 172 patients. Michael's game is a collaborative game that trains the ability to generate alternative hypotheses to the erroneous conclusions that “Michael” draws from common everyday life situations. Results indicated that conviction measured with the 21-item Peters et al. Delusions Inventory improved at the end of the intervention. At 6-month follow-up, the results showed a sustained effect on conviction and a delayed effect on distress and preoccupation for participants in Michael's game compared with participants in the treatment-as-usual condition. Nguyen et al. presented the development of a short, easy-to-use, group-based intervention to improve pleasure and motivation in individuals with schizophrenia, called “Positive Emotion Program for Schizophrenia” (PEPS). A literature review led to the identification of the components of the program, and different beta tests enabled its refinement.

## Conclusion

The 34 papers published on the research topic addressed here highlight the important developments in the field. The variety of technology- and gaming-based solutions proposed across different disorders and sometimes for the same disorders illustrates the high level of creativity that characterizes the field. Such developments are opening avenues for innovation in the assessment, prevention, and treatment of mental disorders.

The first wave of computerized and Internet-based therapies reproduced, in electronic format, previously validated treatments of specific disorders such as anxiety and depression. This first wave tried to overcome the gaps in service provision and use among people with mental disorders [14]. A number of meta-analyses of randomized-controlled trials reported positive outcomes of such treatments for different disorders [15, 16]. Translation of such results in naturalistic settings was, however, compromised by low engagement [17, 18], arguing for a paradigm shift in this area (Fleming et al.).

The present topic provides an overview of possible options and methods to overcome such challenges, including game-based approaches. An important trend is further highlighted: a new generation of computer- and game-based approaches not only reproduces validated face-to-face treatments, but also uses the interaction potential of the technologies to enhance, modify, combine, or create new treatments (Winslow et al.; Zidani et al.; Ben-Moussa et al.; Urech et al.; Riva et al.). Some of these proposed tools include training emerging from developments in cognitive neuroscience (Zidani et al.; Demily et al.; Gaudelus et al.; Vianin). The topic includes articles on schizophrenia and other treatment-resistant conditions. Such papers try to enhance or improve treatments as usual. But again, this observation underlines a new trend. The field initially focused on the service provision gap, but is now working on the improvement of existing treatments or on the development of new treatments.

Some aspects of the developments in the field are still missing in the present topic, particularly those related to the potential addition of machine learning [19, 20]. According to Zhang and Ho, the full potential of e-health, m-health, machine learning, and gaming in psychiatry still needs to be investigated.

In conclusion, this research topic highlights some of the main developments in the field of computers and games that open the horizon for future improvements in tools to enhance mental health and well-being. This growing field offers new intervention tools for the prevention and treatment of a wide range of psychiatric conditions. Although the efficacy of each tool needs to be considered separately, one may expect common factors to specifically contribute to the retention and/or efficacy of some types of interventions. Identifying such common factors will help guide the design of future interventions. Further studies will be needed to assess not only the outcomes of these interventions, but also the processes related to user engagement and associated changes in behavior.

## Author contributions

All authors listed have made a substantial, direct and intellectual contribution to the work, and approved it for publication.

### Conflict of interest statement

SB is now consultant for and owns equities in Cliniques & Développement *In Virtuo*, which distributes virtual environments. The other authors declare that the research was conducted in the absence of any commercial or financial relationships that could be construed as a potential conflict of interest.

## Glossary

***Augmented reality***: The addition of digital components (possibly multiple sensory modalities) to a real-world experience.

***e-health***: The use of the Internet and related technologies for health- and public health-related purposes.

***m-health***: M-health is a component of e-health. The m (for mobility) indicates the use of mobile Internet and related devices (e.g., mobile phones, global positioning systems, patient monitoring devices) for health- and public health-related purposes.

***Serious games***: Games specifically developed to engage users in behaviors with the aim of improving health-related outcomes.

***Virtual reality***: An immersive lifelike experience provided by a computer-simulated interactive environment in which the participant's actions to some extent influence the scenario and the experience.
